# Public Grants for Voluntary Hospitals

**Published:** 1910-10-29

**Authors:** 


### PUBLIC GRANTS FOR VOLUNTARY HOSPITALS.

### I.—A LESSON FROM AMERICA.

The voluntary hospitals, of which this country is so justly proud, have passed through a very remarkable epoch in their history during the past thirty years, and have imperceptibly reached a crisis. At the time of their foundation and until within quite recent years they made no attempt to cope with disease as such, nor to give relief to all the sick persons within their sphere of action. They were for the relief of certain sick persons who had claims upon their supporters or were known to them, and the ticket system was the natural expression of this purpose. Just as benevolent people made choice of those on whom they would confer the blessings of education, and thus kept alive the love of letters in the country during an unlearned age, so the merciful were accustomed to exert themselves to procure for the sick who happened to have a claim upon them the blessings of free treatment. Steadily but surely the conscience of the country has awakened to the fact that education is the birthright of free citizens. It is now stirred to a perception that relief in sickness is a boon which can no longer be bestowed partially on a selected few, but must logically be extended to all who cannot afford to obtain it for themselves. Just as the voluntary schools awoke a demand they could not satisfy, so the voluntary hospitals have in a sense done their work too well; they have made hospital treatment an indispensable part of the duties of the State. Side by side with the voluntary hospital system, but presenting such radical defects as to leave the sphere of the hospitals untouched, a network of rate-supported hospitals has spread itself over the country, and the lesson that every sick person has a claim to relief has been thus legally recognised. But in the process the hospitals have striven desperately to keep pace with the amazing fertility of the race during the period, and have almost unconsciously begun to outstrip the giving powers of the public. Since the care of the sick is now recognised as a national duty, many are beginning to inquire whether the voluntary hospitals have not now performed their work, and may not with advantage surrender their functions to the municipality or State. It is not necessary to recapitulate in this place the weighty reasons for believing such a step would be an irretrievable national calamity. But it is well to probe one cause which seems to propel the more faint-hearted in this direction. It is this. When municipal or State institutions begin to compete with voluntary institutions, the generous supporter of the institution is made to feel the pinch both ways. The rates steadily increase, he perceives that the expenses of the public infirmary make heavy demands on his purse, and he not unnaturally begins to consider whether he can also afford the voluntary subscription. This is the point at which, unless many voluntary institutions are to be slowly undermined, some State or municipal encouragement is urgently demanded.

The American hospital system cannot be compared to our own in detail. But in this particular there is striking resemblance. There are in the United States many institutions which, in their laudable desire to overtake the increase in the suffering population, have outstripped the giving powers of the benevolent. Their work is carried on, as is the case in most British hospitals, with an ever-increasing strain and uncertainty about the funds, and it is evident that without powerful intervention they must close their doors or be absorbed by the public authorities. The intervention they have obtained is that of the State and in some cases that of the municipality.

In the 1910 issue of "Burdett's Annual" there is a very instructive article tracing the course adopted in the various States and in the Canadian provinces in regard to the encouragement of private hospitals. The States are left entirely free to make their own regulations in this particular, and a wide diversity of practice is found to exist. But certain leading principles have slowly been evolved through this very diversity, and as this question is likely soon to become a pressing one in this country much is to be learned from them. There are two obvious dangers to guard against. The first, that State grants extended to voluntary institutions may engender extravagance, and the second that they may tend to discourage private benevolence. The first is obviated by the equitable principle that institutions receiving public appropriations be required to make full financial statements to the public authorities, and be subject so far as the sanitary and economic aspects of their work are concerned to official inspection and investigation. The danger of lessening charitable endeavour is met by the proviso that the State shall in no case pay the entire cost of maintaining any one patient, or more than half the cost of maintaining the hospital. It is found advisable moreover to make no grants for patients for whom the city or State maintains institutions with adequate accommodation.

The various forms in which the pressure on funds of voluntary institutions is eased by public grants are as follows: (1) A yearly sum appropriated for the support of a fixed number of beds to be placed at the service of the municipality; (2) Grants to the hospitals in the nature of a subscription; (3) Grants in aid of purchase of drugs; (4) Grants made to the hospitals on application in consideration of the work performed; (5) State payment *per capita* for free patients after investigation into their circumstances; (6) City grants for partial support of indigent patients; (7) Exemption from taxation; (8) Hospital buildings supplied rent free; (9) Grants for building purposes and maintenance of free patients made after application to the State Board of Public Charities; (10) Grants partly from the State and partly from the municipality for the support of all non-paying patients.

The principle of combining State and municipal aid with private benevolence is now so deeply rooted that it may be said that neither can dispense with the other. Where private philanthropy is absent the work is found speedily to become perfunctory, and the hospitals to be on the whole ill-managed; while in districts where no State encouragement is afforded to institutions for the sick great difficulty is experienced in founding new hospitals, and not seldom those which have been in working order for some time have been compelled to close their doors.

